# The role of gender in Zika prevention behaviors in the Dominican Republic: Findings and programmatic implications from a qualitative study

**DOI:** 10.1371/journal.pntd.0007994

**Published:** 2020-03-06

**Authors:** Tilly Gurman, Anne Ballard Sara, Florentina Villanueva Lorenzo, Desirée Luis, Gabrielle Hunter, Sean Maloney, Ryanne Fujita-Conrads, Elli Leontsini

**Affiliations:** 1 Johns Hopkins Center for Communication Programs, Baltimore, Maryland, United States of America; 2 Universidad Autónoma de Santo Domingo, Santo Domingo, Dominican Republic; 3 Johns Hopkins Bloomberg School of Public Health, Baltimore, Maryland, United States of America; University of Heidelberg, GERMANY

## Abstract

**Background:**

Zika remains an epidemiological threat in Latin America, including the Dominican Republic. Although transmitted by the same mosquito as Dengue and Chikungunya, Zika is unique in the potentially harmful consequences for babies born to women infected during pregnancy. Experts highlight the feminization of Zika, in terms of burden of disease and women’s caregiving responsibilities. Understanding gender’s role in Zika prevention, therefore, is key to strengthening current and future programs.

**Methodology/Principal findings:**

This qualitative study, comprised of 12 focus group discussions and eight in-depth interviews, explored gender’s role in Zika among pregnant and non-pregnant women as well as male partners of pregnant women in the Dominican Republic. Topics included perceptions about Zika and perceived feasibility and effectiveness of prevention behaviors (e.g. cleaning water storage containers, using condoms during pregnancy). Researchers applied grounded theory through a process of deductive coding—classifying data around predetermined categories—followed by inductive coding—identifying themes that emerged from coded data. Study findings uncovered three ways in which gender may influence Zika prevention. First, women are largely responsible for household chores—including cleaning water storage containers—with men as assistants. Second, men described their role in the family as the protector. Finally, men and women believed that partners would perceive suggesting condom use or abstinence during pregnancy as a sign of infidelity.

**Conclusions:**

Current/future Zika programs should address knowledge gaps, especially around water storage cleaning techniques and sexual transmission. Programs should also integrate gender into programming in culturally-relevant ways that avoid reinforcing stereotypes. Furthermore, programs should tailor activities for men, women, as well as the couple. In the end, integrating gender in a way that is mindful of the local context while not exploiting existing gender roles is critical for preventing Zika and similar mosquito-borne diseases, both in the Dominican Republic and throughout the region.

## Author summary

Zika is an important epidemiological threat in Latin America, including the Dominican Republic. Because Zika disproportionately affects women, understanding gender’s role in Zika prevention is key to strengthening current and future programs. We explored the role of gender in Zika among men and women in the Dominican Republic. Topics included perceptions about Zika and perceived feasibility/effectiveness of specific Zika-prevention behaviors. We uncovered that women are largely responsible for household chores—including cleaning water storage containers (a primary mosquito vector habitat)—with men as assistants. In addition, men described their role in the family as the protector. Finally, men and women believed that partners would perceive suggesting condom use or abstinence during pregnancy as a sign of infidelity. Study findings offer valuable programmatic implications that integrate gender-related considerations in social and behavior change programs related to Zika and other arboviruses. In the end, integrating gender in a way that is mindful of the local context while not exploiting existing gender roles is critical for reducing the burden of Zika and other similar mosquito-borne diseases in the Dominican Republic and throughout the region.

## Introduction

### Zika in the Americas

From 2015–2018, the Pan American Health Organization (PAHO) reported 583,451 suspected and 223,477 confirmed cases of Zika in the Americas [1,2]. Zika is a communicable disease mainly transmitted by the *Aedes aegypti* (*Ae*. *aegypti*) mosquito [3]. Although transmitted by the same mosquito as dengue and chikungunya [3], Zika is unique in the potential health and development sequelae in babies born to women infected during pregnancy. These consequences include congenital Zika syndrome which can include neurological birth defects and developmental delays—all of which are still not fully understood [3,4]. Zika can be also be sexually transmitted, further increasing the risk faced by pregnant couples in endemic regions, given that a vaccine and/or treatment does not currently exist [3].

The United States Agency for International Development (USAID) recommends seven Zika-related prevention behaviors that have the greatest potential to reduce Zika transmission and minimize negative pregnancy outcomes [5]. The seven behaviors include two related to personal protection: 1) the use of mosquito repellent and 2) using condoms during pregnancy; three related to household and community vector control: 3) removing standing water, 4) covering water storage containers, and 5) cleaning water storage containers; and two behaviors which enable discussions about Zika risk/prevention with health providers: 6) attending prenatal care visits and 7) seeking counseling about family planning methods [5]. Several of these key Zika prevention behaviors directly relate to gender roles and norms (i.e. condom use during pregnancy, cleaning water storage containers, attending prenatal care visits, seeking family planning counseling).

### Zika in the Dominican Republic

Zika’s epidemiology in the Dominican Republic illustrates feminization of the disease in three aspects [1,6,7]. First, the caseload disproportionately comprises women. Between 2015–2017, the Dominican Republic confirmed a total of 335 Zika cases, 271 of which were among pregnant women [1,6]. This gender disparity in the number of cases is affected by multiple factors, including the fact that the surveillance data comes from the formal health sector, which women are more likely to engage with compared to men. Moreover, Zika testing has been primarily indicated for women who were pregnant or might be pregnant.

Second, challenges related to congenital Zika syndrome likely disproportionately impact women, who often serve as the primary caregivers in households [7]. Between 2015 and 2017, the Dominican Republic documented a total of 85 cases of congenital Zika syndrome [1]. Third, women are often the primary person tasked with household chores, including cleaning responsibilities and managing trash removal [7]. These gender and social norms may directly influence household and community vector control measures to prevent Zika, including whose role it is to clean water storage containers and remove standing water around the home.

To achieve sustained prevention behaviors in homes and communities—given the relatively recent presence of Zika in the Dominican Republic—knowledge of Zika and how to prevent it is an important initial step. Several previous studies have demonstrated high levels of general knowledge of Zika, although specific knowledge about Zika, including knowledge of sexual transmission, is substantially lower [8,9]. For example, a 2017 SMS survey among men and women of reproductive age in the Dominican Republic (n = 1,003) found that 90% of the sample knew that a person can get Zika through a mosquito bite, although only 31% of women and 34% of men knew that Zika could be sexually transmitted [8]. Moreover, only 53% of women and 40% of men knew of the potential sequelae for children born to Zika-infected women [8]. In terms of knowledge of prevention strategies, only 63% of women and 54% of men surveyed were aware of cleaning water storage containers as a Zika prevention strategy and only 17% of women and 20% of men were aware of condoms as a way to reduce the risk of Zika transmission [8].

The fact that reduction of Zika transmission requires individuals to engage in multiple preventive behaviors further complicates prevention efforts. Since the onset of Zika in the Dominican Republic, the Ministry of Public Health and Social Assistance and the USAID have tackled Zika by working to raise awareness and promote specific prevention behaviors at the individual, household, and community levels. Programs aiming to increase Zika prevention behaviors have gone beyond vector control activities and included a range of other interventions, such as mass media promotion, healthcare provider trainings, service delivery protocols, and community mobilization.

### Gender and Zika

Gender affects the risk of exposure to vector-borne diseases, as well as the acceptance and participation in prevention and control programs [10,11]. Gender particularly affects vector-borne diseases that are transmitted in and around homes as gender norms often dictate who is in charge of vector-control activities in the home [10,11]. Integrating a gender perspective into vector-control strategies would allow us to better understand the role of men and women in prevention efforts and better target initiatives [10]. Moreover, gender norms may dictate what are considered men’s or women’s domains, including norms about whether or not men can participate in maternal child health care [12].

Given the disproportionate burden of Zika on women, experts have called for a gendered approach which directly addresses gender roles in society and gender inequalities in health programming [7,13]. To date, however, little research exists about the ways in which gender affects Zika prevention behaviors [13–16]. A recent review of available research literature found that of the 608 publications on Zika, only two examined the relationship between ‘human rights’, ‘gender’ and ‘Zika infection’ [13]. Gender and gender norms may influence the epidemiology of diseases, including Zika, as well as the responses that countries, communities, and families enact to counter disease threats [13]. Disease prevention efforts that fail to address gender may overlook social/structural barriers women and men face to adopting recommended prevention behaviors [13,17].

Previous research has identified ways in which gender roles and related power dynamics can affect the agency of individuals to perform behaviors and should be considered in the formulation of social and behavior change programs [7,12,14–16]. For example, a qualitative study examining patterns of reproductive decision-making among men and women in Peru found that attitudes toward relationships, promiscuity, and sexuality limited women’s ability to initiate discussions on sexual and reproductive health with their partners [15]. These dynamics can affect perceived agency to protect oneself from Zika, including the use of condoms to prevent sexual transmission of Zika during pregnancy [15]. Although some research exists about the role of gender in Zika, there remains a need to further examine the ways in which gender roles and norms may influence perceptions and implementation of Zika prevention behaviors among men and women [16,17]. Better understanding of the gender dynamics inherent in Zika prevention behaviors will better enable Zika programs to develop and deliver effective prevention messages to key audiences.

In addition to the social barriers created by gender roles, government policies and funding have direct ties to the role of gender in Zika. International responses have been critiqued for overlooking the needs of marginalized groups, particularly women, that are most heavily affected by the Zika outbreak [7,13,17]. These critiques point to a pressing need of addressing structural inequalities—including the conditions and contexts of affected populations—in order to tackle global emergencies, such as Zika [13,17,18]. For example, initial Zika-response messages advocating that women delay pregnancy to prevent Zika transmission overlooked the economic, structural, and social barriers women face, including the underlying unmet need for family planning and challenges in accessing family planning services [13].

Experience from past program implementation in Latin America and the Caribbean has also highlighted the need to integrate gender perspectives into Zika research. In the region, many women and girls lack access to basic quality sexual and reproductive health services and more than half of all pregnancies are unintended [14]. Cultural and gender norms can reinforce stereotypical gender and family roles, potentially impacting condom use and perceptions regarding women's role as the caretaker [14,19]. Throughout the region, women spend substantially more time on unpaid tasks which limits their time to focus on professional or personal development and feed into perceptions of women as caretakers [19]. Underlying gender and cultural norms, and a restrictive sexual and reproductive health political landscape, can also lead to a lack of comprehensive public health messaging [14].

Gender inequality remains a persistent and pervasive challenge in the Dominican Republic. For example, the 2015 Gender Gap Index Report—which measures differences in health, education, economy and politics among women and men—ranked the Dominican Republic 86 out of 145 countries [20]. Moreover, approximately 48% of women work in the informal sector, only half of whom report being in control of their income [21]. Additionally, 48% of all pregnancies are unintended [21]. Finally, although intimate partner violence is common in the region, violence against women is on the rise in the Dominican Republic—20% of women experienced violence in 2007 compared to 26% in 2013 [21,22].

The initial international response to the Zika epidemic has been criticized for its failure to acknowledge the impact of gender on disease burden and transmission [13,17,18]. With the sexual transmission of Zika and its potential negative effects on pregnancy outcomes, the burden of disease rests primarily on women and girls of reproductive age [14]. The current study aimed to unpack the influence of gender on Zika prevention behaviors. By examining the ways in which gender may influence Zika prevention behaviors, the study emphasizes the complex social context and enables more gender aware programming in future social and behavior change interventions for Zika.

## Methods

### Ethics statement

Both the Johns Hopkins University Institutional Review Board (JHU IRB) and the Dominican Republic National Center for Maternal and Child Health Research approved this study. Individuals (all of whom were adults ages 18 or older) gave oral consent prior to participating in the study. The researchers used oral consent due to literacy concerns of the population. Both the Dominican Republic and JHU IRB's approved the study using oral consent.

### Study design

The current qualitative study explored perceptions about Zika and perceived feasibility and effectiveness of specific prevention behaviors in the Dominican Republic. The research team, with input and guidance from USAID and local non-governmental partners on the ground, selected Azua and Santo Domingo Este as study sites. Approximately 20% of the population lived below the poverty line in Santo Domingo Este—an urban municipality within the province of Santo Domingo—as compared to 63% in Azua—a peri-urban municipality within the province of Azua [23,24]. The team based its decision on a variety of factors, including the presence of Zika, differences in geographic location (urban and peri-urban, respectively), and stable versus unstable water supply and trash collection, respectively [23,24]. Additionally, Zika prevention programming was being implemented in both study locations.

Community workers from the non-governmental organization Save the Children who were familiar with a particular study site used convenience sampling to recruit the following three types of participants from that site:

1. Pregnant women (ages 18 to 30);
2. Men (ages 18 and over) whose partner was pregnant; and
3. Women (ages 18 to 30) not currently pregnant, with a steady partner and either no children or a single child (considered likely to become pregnant in the near future).

In April 2018, Spanish-speaking researchers conducted 12 focus group discussions (FGDs)—six per region and two per type of study participant—and eight in-depth interviews (IDIs)—four per region, all with men, for a total of 88 participants.

All study participants first engaged in a free listing activity, in which they named all of the behaviors that people in their community perform to prevent Zika. FGD activities addressed Zika risk perceptions as well as perceptions about feasibility and effectiveness of 15 different behaviors, including the above-mentioned seven behaviors for USAID (see Table 1 below). Members of the local research team facilitated the FGDs, all of which took place in community centers. The facilitator led a discussion about whether or not participants perceived that people in their community would deem each behavior feasible or effective. This included substantial discussion and confirmation among participants. After the interactive discussion about each behavior participants voted whether the behavior should be categorized as very, more or less, or not feasible / effective. Final classification was based on the most number of votes. A later activity then asked participants to vote for the three behaviors that they felt people in their community would be most likely to perform.

**Table 1:** Zika-prevention behaviors explored during focus group discussions.

Key Zika prevention behaviors with greatest potential to reduce Zika transmission and minimize negative pregnancy outcomes*	Using mosquito repellent during pregnancy
	Using condoms during pregnancy
	Removing standing water
	Covering water storage containers
	Cleaning water storage containers
	Attending prenatal care visits
	Using family planning
Other Zika prevention behaviors	Using a mosquito net
	Abstaining from sex
	Wearing long sleeves during pregnancy
	Applying bleach on the walls of water storage containers
	Cleaning the *pileta*, a fixed concrete water storage receptacle (Azua only)
	Using screens on windows
	Disposing of tires
	Eliminating rubbish in the home

*Breakthrough ACTION, Breakthrough RESEARCH, U.S. Agency for International Development. Zika Prevention Behavior Matrix. 2018. Available from: https://www.zikacommunicationnetwork.org/sites/default/files/resource_files/Zika-Prevention-Behavior-Matrix-14JUN2018.pdf

In addition, FGDs included an activity where participants simulated the way in which people in their community clean primary household water storage containers. The simulation included work in pairs to list on a flipchart—using drawings and/or words—the materials needed and the step-by-step process for cleaning household water storage containers to prevent Zika. The facilitator then asked for a volunteer pair to demonstrate their cleaning process to the group, providing the pair with the physical materials they identified as necessary. The volunteers simulated cleaning using a large plastic bucket filled with water as a proxy for the *tanque (tank)*, the most common water storage container in both communities. After the step-by-step simulation was complete, the facilitator asked the volunteers clarifying questions and solicited feedback from the larger group. This interactive activity generated discussion about the most common cleaning practices in people’s communities. Each FGD also ended with a conversation regarding gender roles specific to cleaning water storage containers.

IDIs explored men’s perceptions about their community’s current and past level of concern regarding Zika, as well as attitudes about specific Zika prevention behaviors. Men could not participate in both a FGD and an IDI. The IDIs included a more in-depth exploration about a smaller subset of behaviors: using condoms during pregnancy, removing standing water, cleaning water storage containers, permitting vector control workers to enter the home, and attending prenatal care visits. The decision to conduct IDIs with men in addition to the FGDs was to explore in greater detail potentially sensitive behaviors (e.g. condom use) outside of a FGD where social dynamics might bias open responses. The IDIs also allowed more time for in depth discussions regarding men’s perceived role in specific behaviors. During the IDIs, men answered questions in response to a set of photographs, each of which represented a specific Zika-related behavior. Several of the questions focused on the possible influence of gender and social norms. For example, questions asked about what men in their community think about the specific behavior, the role men play, as well as whether that role changes when a man has a pregnant partner.

Eligible individuals—all of whom were legal adults—who chose to participate in the study provided informed oral consent prior to beginning the first research activity (free listing). In order to assure consistency in the consent process, interviewers used a standardized oral consent script, approved by the IRB. Interviewers only allowed individuals that provided oral consent to continue on as study participants. In other words, individuals documented as participating in the free listing research activity represented those that had provided oral consent. Study participants received a basket of basic cleaning materials for their time and were reimbursed for transportation expenses.

Data analysis, which used a Grounded Theory approach of both deductive and inductive reasoning, occurred in three steps [25]. First, two researchers independently used Atlas.ti software to code Spanish transcripts, according to a set of general categories finalized during data collection. The researchers initially double-coded 20 percent of the transcripts in order to ensure consistency between coders. Second, a deeper inductive analysis of the coded transcripts took place approximately one month later over a five-day period. This second step comprised a data analysis team of four researchers involved with data collection and two local partners familiar with Zika in the Dominican Republic. During this step, the data analysis team performed axial coding whereby they reviewed the deductively coded data and worked together to identify relationships between the initial codes, relate them to one another, and identify core themes that emerged. The data analysis team presented findings from the first two steps during an in-person workshop with external partners, including representatives from USAID and non-governmental organizations engaged in Zika-prevention activities in the Dominican Republic. Workshop participants provided feedback on the findings and offered insight that informed the development of practical and actionable evidence-based recommendations. The final step of the data analysis process involved the study’s first two authors’ further reviewing the themes and coded data with a gender lens.

## Results

### Study participants

The study engaged a total of 88 participants, 86 of whom completed both the free listing activity and either a FGD or an IDI. Participants ranged in age from 18 to 47 (mean: 24.3) and had 1.2 children. Among study participants, 40% worked in the home and 59% had completed at least some level of secondary education. As previously stated, study eligibility allowed for women ages 18 to 30 and men 18 and above. (See Table 2 below for breakdown of participants by region.)

**Table 2:** Study participants engaging in either a focus group discussion or in-depth interview, by type of participant and region.

Type of participant	Number of participants, per region		Total number of participants
	Santo Domingo Este	Azua	
Male partners of pregnant women	14	18	32
Pregnant women	16	11	27
Non-pregnant women	14	13	27
Total number of participants	44	42	86

### Perceptions about Zika and prevention behaviors

In the individual free listing activity—conducted immediately prior to the IDI/FGD—participants (n = 88) offered 317 responses (mean = 3.6 responses per participant) to the question about what people in their community do to prevent Zika. These responses represented 24 distinct behaviors (see Table 3 for the behaviors receiving at least 10 mentions). No single behavior received mentions by more than half of the 88 participants. The most frequently reported behavior was the elimination of rubbish in the community and/or around the home, closely followed by adding bleach to water. Condom use only received 12 mentions (9 women; 3 men) and no participant mentioned seeking prenatal care or using family planning as a Zika-prevention behavior performed by people in their community.

**Table 3:** Zika prevention behaviors carried out in the community mentioned by at least 10 participants during the free listing activity (n = 88).

Behaviors mentioned	Number of mentions		
	Total	Men	Women
Eliminating rubbish in the community and/or around the home	37	10	27
Adding bleach to water	32	13	19
Cleaning water storage containers	30	8	22
Covering water storage containers	28	5	23
Eliminating/avoiding standing water	25	9	16
Cleaning (in general)	20	6	14
Practicing good hygiene behaviors	16	9	7
Getting a vaccine	14	6	8
Using a mosquito net	13	4	9
Using condoms during sex	12	3	9
Applying bleach to water storage containers (no specification as to how applied)	10	3	7

From the IDIs and FGDs, most participants saw the link between mosquitoes and Zika. Fewer, however, understood the possibility of sexual transmission of Zika. For example, one pregnant woman commented when assessing the effectiveness of using condoms during pregnancy to prevent Zika, *“That doesn’t do anything for Zika… Zika is not a disease that is transmitted sexually*.*”* Moreover, some participants connected dengue, but not Zika, to certain vector control behaviors. For example, when discussing cleaning water storage containers to prevent Zika, one non-pregnant woman commented, *“That’s for mosquitoes so that they do not become larva in the water*. *Then they wash the tank with bleach and then pour some in the water for mosquitoes for dengue*. *That’s not for Zika*.*”* These general findings were similar across study sites and regardless of gender.

When participants were asked to individually select the three behaviors they believed that people in their community would be most likely to do, several notable differences surfaced between men’s and women’s total numbers (see Table 4). Although vector control behaviors received a higher number of votes among both men and women, applying bleach to container walls received a higher number of votes among pregnant and non-pregnant women compared to men. Among men, removing standing water at home received the greatest number of votes, followed by three vector control behaviors—covering water storage containers, cleaning water storage containers, and eliminating rubbish—tying for second place. Additionally, attending prenatal care visits ranked fourth for men, whereas this behavior did not rank in the top five among women, receiving only three votes among pregnant women and three votes among non-pregnant women.

**Table 4:** Top behaviors people in communities were most likely to do, as voted by focus group discussion participants.

Rank*	Men	Pregnant Women	Non-pregnant Women
1	Removing standing water at home	Covering water storage containers	Using bleach on water storage container walls
2	Covering water storage containers	Using bleach on water storage container walls	Covering water storage containers
	Cleaning water storage containers	Removing standing water at home	
	Eliminating rubbish		
3	Using a mosquito net	Cleaning water storage containers	Cleaning water storage containers
4	Eliminating tires	Eliminating rubbish	Using a mosquito net
	Attending prenatal care visits		
5	Using bleach on water storage container walls	Eliminating tires	Removing standing water at home

* **Note:** Rank was based on the total number of votes received during the activity. Behaviors that received the same number of votes are included under the same rank.

### Gender roles in Zika prevention behaviors

Although many of the general perceptions about Zika did not vary much by gender, when discussing Zika prevention behaviors, the role of gender surfaced in three ways. First, household cleaning chores were primarily seen as the woman’s domain, although men may assist. Second, most men saw themselves as the protector of their family, including that of their pregnant wife and unborn child. Third, many men and women connected condom use and abstinence to lack of trust between partners and perceptions about suspected infidelity.

#### Household cleaning chores as the woman’s domain

In general, both men and women remarked that women were the ones primarily responsible for household chores, including cleaning water storage containers. The majority of women noted that they were primarily responsible for tasks related to cleaning around the home as seen through this exchange of non-pregnant women,

***Facilitator:*** *In the community who is generally responsible for this type of activity,*

                              *cleaning a water storage container?*

***Participant 6:*** *Women.*

***Participant 5:*** *Women.*

***Participant 2:*** *Men too when the woman is not home. Mainly it’s the woman.*

***Participant 6***: *I have never seen a man cleaning a water storage container.*

***Participant 2***: *It’s just the women.*

***Participant 3***: *Primarily it’s us.*

***Participant 4:*** *No, there are men who for wanting to help will do whatever [cleaning] but mainly it’s the women …There are men who think this is something of the house [housework], the cleaning responsibilities of women. You know machismo men.*

***Participant 5:*** *The only thing men do is dump the trash….*

Several men brought up the socially constructed male gender roles (*machismo*) in the community and the commonly accepted perception that household work is the woman’s responsibility. One noted, “*Here they are so machista that women have to do all their work no matter what*.” Another participant concurred stating,

*The woman is the most important in the Dominican family because the woman is the hidden brain behind the stupid truth. The woman knows …she can carry the day to day of the house. Mentally the man does not have this. We only have the money, nothing else*.

Several male and female respondents, nevertheless, described men’s role as that of a collaborator/assistant. With a pregnant partner, men may become more engaged. One man declared, *“It is the woman who [cleans water storage containers]*, *but we have men that nowadays are in the home and have to do it*. *We also do it*, *we as men need to help*.*”* Another man shared how the role of the man changes with a pregnant partner, commenting, *“Because the woman with a belly [pregnant] cannot move much*, *cannot strain herself*, *cannot carry water*. *So*, *the role of the man changes there*, *because he has to do these chores*.*”* He later described how the role further shifts later in the pregnancy, stating,

*When the woman is pregnant and is early on in the pregnancy, she can do household chores. But when she is further along, like eight or seven months… it changes because she feels exhausted due to the belly. So there the man has to go and help her more*.

Some women similarly talked about their partners helping them sometimes with cleaning responsibilities, especially during pregnancy. For example, one pregnant woman declared about house cleaning chores during pregnancy, *“One largely does everything*, *but when there are heavy things*, *they [men] help*.*”* Other women talked about challenges with men helping out because they may not be around much, either due to work or other demands on their time. For example, one pregnant woman noted, “*Men right now* …*they just go to the house to eat*.” Another woman continued the conversation stating, many men say “*I’m not a woman*. *Am I a woman [that should be] washing a water container or doing whatever in the house with a rag*?” In another FGD, several women also critiqued that some women become useless and lazy during pregnancy. One pregnant woman stated, *“There are some who all they want to do is lie down*. *Not me*. *With my belly I still do everything*. *I carry water*, *I do everything*.*”*

#### Men as protector of the family

In addition to viewing themselves as assistants to women for household chores, most men talked about a central role they play of protecting their family. The man that previously talked about how men’s role changed during their partner’s pregnancy, especially later in pregnancy, commented, *“It’s like I told you earlier about taking care of her*, *taking care of the pregnancy*, *that she [partner] doesn’t overexert herself*, *that she doesn’t walk too far*.*”*

Respondents also mentioned this role of protector when discussing attending prenatal care visits. Quite a few men commented on wanting to accompany their partners at their prenatal care visits, while noting, on the other hand, that accompanying women is not common practice. For example, one man remarked,

*Well in my case I always [ask] my wife if she wants me to accompany her, because if she wants me to go with her, or we go together, or I go and drop her off…But here in this town that is not very common. Women always go alone…sometimes because of work and sometimes not. Me, in my case, I always would like to be there, because, look, at least I went when she got the sonogram. I went. I go, I go, because you have to support*…

Another man similarly perceived that most men in his community do not accompany their partner to prenatal care visits. He provided a counter example when he described his own behavior, stating, *“I go with mine*, *not because I feel that it is an obligation*, *but rather because I want to nourish myself*, *because I am new at this*, *it is my first child and I want to know everything* …*” Some m*en also discussed how men become more concerned with their partner during pregnancy. For example, one man described this shift, stating, *“The man is a person who thinks a lot in the health of the woman and the baby you’re waiting for and it’s something they do very voluntarily*.*”*

Although participants explained that men may want to accompany their pregnant partners to prenatal visits, participants, on the flip side, provided various reasons why a man would not accompany their pregnant partner to prenatal care visits. Men and women discussed challenges that may impact men’s ability to attend appointments, including men not being able to take time away from work as well as the long wait times. For example, when describing why attending prenatal care visits was not easy, one man stated,

*It’s a good prevention measure [receiving prenatal care] but it’s not easy…You go to the hospital at five in the morning to get a ticket and they are already gone, so your wife can’t get checked …Another thing is that the doctors arrive three or four hours later [after you arrive] so you leave around three or four in the afternoon*.

Furthermore, a common belief—whether self-perceived or resulting from actual experience—was that a man was not allowed or welcomed to attend prenatal care appointments. When asked whether men are allowed to participate in prenatal visits, one interview participant described his experience, stating, "*Here in the hospital in Azua only women enter*.*”* He continued by saying that although he has accompanied her to her visits, he had not entered the consultation room. He further clarified, “*I always go*, *but I’ve never gone inside*, *but they’ve never told me to come in*.” He later highlighted another belief that going to the doctor, including prenatal care, is a woman’s issue and that men just do not have the habit of going to the doctor.

In addition to prenatal care, the discussion around condom use highlighted many men’s desire to protect their family—including both their partner and baby. For example, one participant stated, *“I am thinking that it is good to use it [a condom] because in order to… protect my partner and my child that is on the way*, *I should use it*.*”* On the other hand, the argument of protecting one’s family was sometimes presented as the rationale for not using a condom. For example, a participant who earlier in a FGD commented on his willingness to use a condom with his partner to not transmit Zika, later questioned using condoms because, *“The lubricant that it [the condom] brings is what is harmful*.*”* Other men concurred noting, “*There are women that would get hurt by it [condoms] inside*. *There are women who can’t use it* …*They [condoms] bring diseases down there because it [contains] an oil*.” Another man noted the lubricant on the condom can speed up the heart of the woman, potentially hurting her. Women did not seem to have this similar concern about harm from using condoms.

#### Condom use, abstinence, and their connection to trust and infidelity

Many men and women alike noted that most partners do not see the purpose in using a condom during pregnancy because the woman is already pregnant. Most men and women linked trust among couples to condom use, expressing that partners that don’t use condoms trust one another. Moreover, condom use during pregnancy elicited concerns about infidelity and, as a result, was not top of mind in relation to Zika prevention. For example, while reflecting on what a man would think in regard to using a condom, one pregnant woman expressed, *“…Why would you want to use a condom with me if you are my woman and you live inside my house*? *You’re doing something outside [the house]*. *That’s what most men here think*.*”* In another FGD with non-pregnant women one woman shared,

*Ninety percent of men are unfaithful. It’s more like ninety-nine percent. And if they leave and have an adventure [affair] with a woman who is infected with Zika, he could bring it back to the house. But if his wife is waiting for him with one [a condom] it’s very unlikely he will use it*.

Similarly, several non-pregnant women in a FGD stated the following, in response to whether couples would use condoms during pregnancy,

***Participant 8:*** *No. Because that is not something that I have in mind. Very few people [do].*

***Participant 6:*** *Oh, I have it in mind.*

***Facilitator:*** *How?*

***Participant 3:*** *You have to use it [condoms] with your partner with this stuff that exists in these times*.

***Participant 7***: *You have to use a condom and even with one’s partner you have to use it to prevent many illnesses on the street that exist because men do not have a flag [insinuating infidelity].*

When most men and women talked about not using a condom during pregnancy, they also often introduced the concern about trust between partners, perceptions or experiences of infidelity—since there is no need to use condoms to avoid pregnancy—and preserving the relationship. For example, one man noted, “*I use it [condom] for precaution*. *But with my wife I don’t use it [condom]*.” Additionally, when asked by the facilitator about the feasibility of using a condom during pregnancy, several men in a FGD stated,

***Participant 6:*** *It's difficult, very difficult.*

***Participant 7:*** *It is difficult because once she is already pregnant by someone how is that man going to use a condom after the woman is pregnant by you…*

***Participant 2:*** *That depends. If, for example, your woman is already pregnant and, after she got pregnant, you come and get sick from Zika, now you have to use a condom to avoid getting her sick.*

***Participant 5:*** *Yes, I agree with you. But to preserve the marriage, it is better not to use it. Why? Because you are going to go now that your wife is pregnant, and you tell her, “we are going to use this condom” she is going to tell you, “Who have you been with? Where were you?” And she’s not going to want to have anything to do with you… So that’s what I mean that it’s very difficult.*

***Participant 3:*** *It’s difficult because it’s going to [create] contradictions.*

The desire to protect one’s partner, on the other hand, was sometimes connected to infidelity. In one FGD, men supported the perception that using condoms is associated with infidelity by noting that it is common for men to be unfaithful and condoms should be used to prevent the sexual transmission of diseases and infections to their spouse or primary partner. For example, in another FGD, two men stated,

***Participant 5:*** *It [condoms] really works because sometimes you’re on the streets and you behave in a different way than you do at home, you understand me, and any bacteria or anything can be prevented.*

***Participant 4:*** *Yes, it really is like he says because we are really men and we hang out on the street. We can make mistakes and we can fall into something, which one does not want at that moment and fall. And when we arrive at the house, for example, to your woman, and you have to think.*

Abstinence during pregnancy was also often linked with concerns of infidelity. For example, one non-pregnant woman noted, “*You can’t [practice abstinence]* …*when a woman is jealous they think ‘you have another woman’*. *If the woman gets like that*, *the man will never understand*. *Men never understand that part*.” Another participant agreed, following her answer by stating, "*If someone says that*, *that they don’t have to have sexual relations*, *the man is brutal*. *The man will say to you ‘no*, *you’re cheating on me*. *I’m going to look for another [woman] on the street*.*’”*

## Discussion

Our study uncovered important community perspectives related to Zika prevention. Similar to previous studies, men and women in the current study recognized the link between Zika and mosquitoes, yet not necessarily the possibility of sexual transmission of Zika [7,8,14]. Men and women also equally stressed the value of having a clean home and the importance of cleaning water storage containers to prevent Zika. At the same time, men and women often talked in more general terms and did not describe correct Zika-specific techniques for elimination of mosquito habitats. As highlighted above, the current study also uncovered important gender considerations regarding Zika prevention behaviors among men and women in the Dominican Republic.

While previous literature linking gender and Zika largely centers around reproductive and sexual health [14,15], the current study adds to this existing body of literature by providing evidence that gender roles and norms may influence the practice of vector control behaviors for Zika. Current study findings support previous literature which stresses that, regardless of employment status, women are largely responsible for household chores [7,10,11]. Study findings uncovered similar beliefs around gender roles specific to cleaning, although both men and women saw men as potential collaborators. In particular, participants stated that when the woman is pregnant—especially later in pregnancy—the man may assume a more active role in washing water storage containers. Men’s motivation to protect one’s family also surfaced as a prominent theme during the discussions about cleaning water storage containers as well as attending prenatal care appointments and condom use during pregnancy. The above findings stress that Zika prevention interventions should aim to expand upon men’s willingness to help their partner and have them see themselves as co-responsible in preventing congenital Zika.

Over 90% of women in the Dominican Republic attend at least four prenatal care visits [21]. Given this reality, one vital ongoing opportunity to engage men and women in discussions about men’s role is during prenatal visits. Prenatal care visits can provide a potentially neutral environment for discussing ways that men can take a more active role during their partner’s pregnancy to ensure an environment free from mosquitoes, and, therefore, reduce the risk of congenital Zika. Such an approach would be critical if the goal is to create programs that take a gender-transformative approach to addressing gender considerations related to Zika [26].

Both men and women perceived condom use as unnecessary during pregnancy, due to the fact that the woman is already pregnant. This rationale overlooks the Zika-prevention aspects of using condoms. Although men and women expressed that using condoms during pregnancy was not only unnecessary but also uncommon, some men did express a willingness to use condoms during pregnancy if they knew it could protect their family.

Condom use during pregnancy, more importantly, was similarly linked with perceptions of infidelity. Although there was some level of acknowledgment among men and women that using condoms during pregnancy could help prevent sexual transmission of Zika, the fear of being accused of infidelity by their partner was present and seemed to inhibit partners from negotiating condom use during pregnancy. These findings about infidelity connotations of condom use during pregnancy mirrors what has been seen elsewhere in Latin America [14,15].

Approaching any health topic, including Zika, with a gender lens extends beyond gender dynamics and roles within households and into the health system which serve women of reproductive age [14]. Moreover, ensuring greater gender equity in health requires meaningful engagement of men and, in order to do so, it is critical to consider the ways in which institutions and policies may influence that engagement [27]. For example, current study findings suggest that although it would be important to motivate men to join prenatal visits with their partner—appealing to the importance they place to protecting their family—programs must simultaneously create a clinical service environment that welcomes male partners. Previous studies in Latin America have similarly found that men have expressed feeling excluded from reproductive decision-making in hospitals and attending prenatal visits [14,15].

The current study experienced two limitations. First, the study only included participants from two regions in the Dominican Republic. Due to financial and time constraints, the study was unable to include participation from additional regions. At the same time, these two regions were some of the most hard-hit areas of Zika, and the study does not claim to be representative of all the Dominican Republic. Similarly, due to financial and time constraints, the study was not able to conduct IDIs with women. IDIs with men were prioritized to specifically expand on issues related to Zika prevention and gender, and explore potentially sensitive behaviors regarding men’s perceived role in specific behaviors. Second, the study explored participants’ perceptions about what their community believes, not whether the participants actually perform the behaviors. This projective technique was used to enable participants to express their opinions and beliefs openly, even if not directly disclosing their own personal behavior.

In spite of the above limitations, the current study provides four general recommendations for future programs. First, programs should continue to address Zika knowledge gaps, especially around water storage cleaning techniques and sexual transmission of Zika. Building on the value men and women place on cleanliness, future programs should emphasize and distinguish between general cleaning and the elimination of mosquito reservoirs. In particular, details about the function of bleach to kill mosquito eggs attached to water container walls and the exact bleach application technique required to be effective would be especially useful [5]. In addition, in order to convince couples to use condoms during pregnancy, they must first make the connection between sexual intercourse, Zika transmission, and the potential consequences on the fetus. Although necessary, however, knowledge alone is not sufficient. Therefore, once couples believe that Zika can be sexually transmitted, programs can begin to tackle the gender roles that affect condom use during pregnancy and address myths, beliefs, and misconceptions about infidelity and condom use.

Second, programs should reflect upon how they can appropriately incorporate the role of protector that men perceive about themselves. For example, programs could promote involving men in vector control behaviors (e.g., water storage container cleaning) especially during pregnancy. Similarly, programs could frame using condoms during pregnancy as something that a man can do to protect his family. In addition, health care facilities should implement strategies that increase partner participation and create a more welcoming environment, such as having men play a specific role at prenatal care visits, expanding clinic hours so that men’s work schedules do not conflict with appointments, and reducing wait time at prenatal care appointments. Normalizing couples’ attending prenatal care visits together can facilitate more open discussion about critical Zika prevention behaviors during pregnancy that benefit and require participation of both partners.

It is critical, however, when programs incorporate research insights to encourage male involvement and build upon the fact that men view themselves as their family’s protector, that they avoid reinforcing stereotypes. Instead of reinforcing a potentially negative power imbalance between men and women, programs could, instead, frame men’s beliefs from the perspective of a couple working together to protect their family. Programs also need to consider the most appropriate way to integrate male involvement and manage sensitive conversations among couples in contexts such as the Dominican Republic where intimate partner violence is prevalent and increasing [21]. The Gender Integration Continuum posits the importance of programs’ gaining awareness of the role that gender plays in health and striving to transform gender roles in their work [26]. Such a framework can provide useful guidance for programs wanting to identify ways to address gender in a more mindful way.

Third, programs promoting condom use during pregnancy must be more gender- aware in their framing of messages and provision of services. Prenatal care visits can provide a good opportunity for providers to discuss condom use as a routine practice during pregnancy—a short-term action to help prevent congenital Zika. By creating a neutral space for couples where men are made to feel welcomed, health facilities, can work to decrease stigma and associations of using condoms during pregnancy and reduce the association between condom use and infidelity. As a result, these discussions could eliminate some of the obstacles pregnant women may face in negotiating condom use with their partners. The creation of prenatal care services that are welcoming to men can further help to ensure that couples attend services together. It is through meaningful engagement of men during these encounters that programs can continue to ensure greater gender equity [27]. Such movement towards greater gender equity may help promote more equality in sexual health decision-making around using condoms during pregnancy to prevent Zika.

Finally, Zika-related programming should be multi-faceted, including components tailored for men alone, for women alone, as well as for the couple as a unit. For example, given women's sometimes limited power to make reproductive health decisions in the home, interventions tailored to men is paramount [16]. In particular, programs may want to create tailored messages to convince men about the long-term benefits of the short-term practice of using condoms during pregnancy. Similarly, the couple should be the focus of other tailored messages, in order to recognize that the responsibility of reproductive health is in the hands of both men and women—not women and girls alone [7,16].

In conclusion, the current study contributes to an important step in gender- aware programming—critically examining gender roles, norms, and dynamics [26]. Given that the list of sequelae of Zika is evolving as the scientific and medical community continues to learn about the impact of congenital infection [4], the need to continue promoting Zika prevention behaviors remains relevant. Critically examining the ways in which gender roles may affect Zika prevention behaviors is essential if current and future programs wish to reduce the burden of Zika and other similar mosquito-borne diseases in the Dominican Republic and throughout the region [13]. Zika programs that strategically work towards integrating gender into its efforts will not only challenge gender inequalities and restrictive norms but may ultimately be more effective in achieving long-term and sustainable outcomes.
